# Ceramide Analysis in Combination With Genetic Testing May Provide a Precise Diagnosis for Self-Healing Collodion Babies

**DOI:** 10.1016/j.jlr.2022.100308

**Published:** 2022-11-01

**Authors:** Takuya Takeichi, Yusuke Ohno, Kana Tanahashi, Yasutoshi Ito, Ken Shiraishi, Ryo Utsunomiya, Satoshi Yoshida, Kenta Ikeda, Hayato Nomura, Shin Morizane, Koji Sayama, Tomoo Ogi, Yoshinao Muro, Akio Kihara, Masashi Akiyama

**Affiliations:** 1 Department of Dermatology, Nagoya University Graduate School of Medicine, Nagoya, Japan; 2 Faculty of Pharmaceutical Sciences, Hokkaido University, Sapporo, Japan; 3 Department of Dermatology, Ehime University Graduate School of Medicine, Ehime, Japan; 4 Department of Dermatology, Okayama University Graduate School of Medicine, Dentistry, and Pharmaceutical Sciences, Okayama, Japan; 5 Department of Genetics, Research Institute of Environmental Medicine (RIeM), Nagoya University, Nagoya, Japan; 6 Department of Human Genetics and Molecular Biology, Nagoya University Graduate School of Medicine, Nagoya, Japan

**Keywords:** acylceramide, lipids, epidermal ceramides, CYP4F22, skin barrier, stratum corneum, tape stripping, autosomal recessive congenital ichthyosis, fatty acid ω-hydroxylase, congenital ichthyosiform erythroderma, ARCI, autosomal recessive congenital ichthyosis, CLE, corneocyte lipid envelope, SHCB, self-healing collodion baby, ULC, ultra long chain, WES, whole-exome sequencing

## Abstract

Self-healing collodion baby (SHCB), also called “self-improving collodion baby”, is a rare mild variant of autosomal recessive congenital ichthyosis and is defined as a collodion baby who shows the nearly complete resolution of scaling within the first 3 months to 1 year of life. However, during the neonatal period, it is not easy to distinguish SHCB from other inflammatory forms of autosomal recessive congenital ichthyosis, such as congenital ichthyosiform erythroderma. Here, we report a case study of two Japanese SHCB patients with compound heterozygous mutations, c.235G>T (p.(Glu79∗))/ c.1189C>T (p.(Arg397Cys)) and c.1295A>G (p.(Tyr432Cys))/ c.1138delG (p.(Asp380Thrfs∗3)), in *CYP4F22*, which encodes cytochrome P450, family 4, subfamily F, polypeptide 22 (CYP4F22). Immunohistochemically, inflammation with the strong expression of IL-17C, IL-36γ, and TNF-α was seen in the skin at birth. CYP4F22 is an ultra-long-chain FA ω-hydroxylase responsible for ω-*O*-acylceramide (acylceramide) production. Among the epidermal ceramides, acylceramide is a key lipid in maintaining the epidermal permeability barrier function. We found that the levels of ceramides with ω-hydroxy FAs including acylceramides and the levels of protein-bound ceramides were much lower in stratum corneum samples obtained by tape stripping from SHCB patients than in those from their unaffected parents and individuals without SHCB. Additionally, our cell-based enzyme assay revealed that two mutants, p.(Glu79∗) and p.(Arg397Cys), had no enzyme activity. Our findings suggest that genetic testing coupled with noninvasive ceramide analyses using tape-stripped stratum corneum samples might be useful for the early and precise diagnosis of congenital ichthyoses, including SHCB.

The permeability barrier function of the stratum corneum (outermost layer of the epidermis) is achieved through the integration of lipids and proteins in the terminally differentiated keratinocytes (corneocytes); the cell membrane is replaced by corneocyte lipid envelope (CLE) mainly comprised of protein-bound ceramides, and the space between the cells is filled with multi-laminar lipid lamellae composed of ceramides, cholesterol, and free FAs (1). Among the elements of the barrier structure in the stratum corneum of human skin, the lipid lamellae and CLE are of critical importance to skin barrier function (2). The lipid lamellae and CLE are essential for the integrity of the permeability barrier, and these lacks are a major structural defect behind many diseases of barrier function (3).

Each ceramide class is named using a combination of abbreviations for the constituent FAs (N, non-hydroxy FA; A, α-hydroxy FA; O, ω-hydroxy FA; EO, esterified ω-hydroxy FA; P–O, protein-bound ω-hydroxy FA) and long-chain bases (S, sphingosine; DS, dihydrosphingosine; P, phytosphingosine; H, 6-hydroxysphingosine; SD, 4,14-sphingadiene ) (supplemental Fig. S1) (4). EO ceramides (EOS, EODS, EOP, EOH, and EOSD) are referred to as ω-O-acylceramides (acylceramides), and a linoleic acid is the predominant FA in the ω-position of these ceramides (4). In humans, the major acylceramide classes are EOS, EOH, and EOP, in order of abundance, while EODS and EOSD are barely detectable (4, 5). Acylceramides are essential for the formation and maintenance of lipid lamellae (6, 7). Conventional ceramides (N and A ceramides) have FAs with a chain length (C) of C16–C24 in most tissues and C16–C28 in the epidermis, whereas acylceramides have much longer FAs (C30–C36) (4, 5). FAs are classified into long-chain FAs (C11–C20), very-long-chain FAs (≥C21), and ultra-long-chain (ULC; ≥C26) FAs (8).

A crucial step in acylceramide synthesis is the hydroxylation of the ULCFA at the ω-position by the ULCFA ω-hydroxylase CYP4F22 (cytochrome P450, family 4, subfamily F, polypeptide 22) (9, 10). *CYP4F22* has been identified as a causative gene of autosomal recessive congenital ichthyosis (ARCI) (11). ARCI is an umbrella term used to describe a cutaneous phenotype of erythema and scaling over almost the entire body at birth (3). To date, 57 pathogenic mutations in *CYP4F22* have been reported in ARCI, including those causing self-healing collodion baby (SHCB) (www.hgmd.cf.ac.uk, Human Gene Mutation Database Professional, as of 2021.4) (12). Of these, 17 missense mutations and one frameshift mutation were examined for their effect on activity of CYP4F22 (9, 13). CYP4F22 deficiency leads to defective acylceramide synthesis, resulting in reduced amounts of protein-bound ceramides and the malformation of the CLE in the stratum corneum. These ceramide abnormalities cause impaired stratum corneum barrier function in ARCI and SHCB.

An SHCB, also called “a self-improving collodion baby”, is characterized as a collodion baby with the nearly complete resolution of scaling within the first three months to one year of life (11). Thus, SHCB seems to be a relatively mild type of ARCI. However, a quality-of-life survey by Hake *et al.* concluded that SHCB is a milder, underestimated, clinical variant of ARCI that includes distinct features such as brachydactyly and ear kinking (14).

Recently, Mohamad *et al.* reported 62 Middle Eastern families of various ethnic backgrounds with ARCI (15). In their paper, pathogenic variants were identified by whole-exome sequencing (WES) in most ARCI-associated genes, including *TGM1* (21%), *CYP4F22* (18%), *ALOX12B* (14%), *ABCA12* (10%), *ALOXE3* (6%), *NIPAL4* (5%), *PNPLA1* (3%), *LIPN* (2%), and *SDR9C7* (2%) (15). In 19% of the ARCI cases, no mutation was identified (15). Most of the *CYP4F22* mutations in their cohort resulted in congenital ichthyosiform erythroderma (15). Another recent study reported on genetic analyses performed using different sequencing methods, including Sanger sequencing or next-generation sequencing, for 68 patients with the clinical diagnosis of ARCI, including 16 SHCB patients (14). Most of the causative mutations in the ARCI cohort were found in *TGM1* (27.9%), followed by *ALOX12B* (16.2%), *ALOXE3* (14.7%), *NIPAL4* (13.2%), *ABCA12* (13.2%), *PNPLA1* (7.4%), *CYP4F22* (5.9%), and *SDR9C7* (1.5%) (14). The genetically confirmed SHCB patients presented causative mutations in *ALOXE3* (50.0%), *ALOX12B* (37.5%), *PNPLA1* (6.3%), and *CYP4F22* (6.3%) (14).

At birth, it cannot be determined whether collodion babies will become self-healing or will develop congenital ichthyosiform erythroderma, a severe form of ARCI. If the collodion baby phenotype is not self-healing and the patients develop congenital ichthyosiform erythroderma, then the itching, pain from fissures, and other symptoms are lifelong. Thus, it is very important for the families to know whether the phenotype is SHCB. Collodion babies with mutations in other genes causative of ARCI, such as *ABCA12*, are not expected to be self-healing. Therefore, detecting mutations in *CYP4F22* and corresponding ceramide abnormalities would give us supportive data for the early diagnosis of SHCB. Here, we describe two Japanese SHCBs with compound heterozygous mutations in *CYP4F22* from two independent families. The findings obtained in the present study suggest that genetic testing in combination with the analysis of ceramides from the stratum corneum in ARCI patients might be useful for the early and precise diagnosis of SHCB.

## Materials and Methods

### Ethics

This study was approved by the ethics committees of Nagoya University Graduate School of Medicine (Permit nos.: 2013-0279 and 2016-0412) and Hokkaido University (Permit no.: 2020-002). Informed consent was obtained from all volunteers and the guardians of an SHCB patient, and the research was conducted in accordance with the principles expressed in *the Declaration of Helsinki*.

### Whole-exome sequencing

Exonic DNA was captured using the Agilent SureSelect Human All Exon v5 target enrichment kit (Agilent Technologies, Santa Clara, CA), and sequencing was performed with paired-end 150-bp reads on the Illumina Hiseq 2500 (Illumina). About 150 M reads/individual were generated, resulting in approximately 100x coverage of the targeted exome. The Genome Analysis Toolkit (GATK v3.5) (Broad Institute) was used to perform variant discovery and genotyping. SNPs and indels were named according to GATK best practices. Variants were filtered under the assumption of a recessive inheritance model.

### Genotyping of ten reported *FLG* mutations in the Japanese population

Real-time PCR-based genotyping of the *Filaggrin* (*FLG*) mutations was performed with the TaqMan probe genotyping assay, which we established in a previous study (16).

### Immunohistochemical analyses

Immunohistochemical analysis of skin samples from one of the participants (case 1) was performed as described previously (17), with slight modifications. Thin sections (4 μm) were cut from samples embedded in paraffin blocks. The sections were soaked for 20 min at room temperature in 0.3% H_2_O_2_/methanol to block endogenous peroxidase activity. After being washed in PBS with 0.01% Triton X-100, the sections were incubated for 30 min in PBS with 4% bovine serum albumin, followed by incubation overnight with the primary antibodies, polyclonal rabbit anti-IL-17C antibody (bs-2611R, Bioss, Woburn, MA; dilution 1:1000), anti-IL-36γ antibody (ab156783; Abcam, Cambridge, UK; dilution 1:1000), and anti-TNF-α antibody (bs-2081R; Bioss; dilution 1:1000) in PBS containing 1% bovine serum albumin. After being washed in PBS, the thin sections were stained with Dako EnVision+Single Reagents (HRP, rabbit) (Agilent Technologies, Santa Clara, CA) for 30 min at room temperature. An Olympus BX51 (Olympus Corporation, Tokyo, Japan) was used for photography.

### Tape stripping and lipid analysis by LC/MS/MS

To examine the ceramide species present in the stratum corneum, tape stripping was performed by pressing and stripping an adhesive acrylic film (465#40; Teraoka Seisakusho, Tokyo, Japan) on the skin of the right leg of case 1, case 2, and both parents of case 1. Samples were also taken from the right leg of five normal children (around 2 years after birth) as controls. Five strips measuring 25 × 50 mm each were obtained from a single individual. The second strip was cut to 10 × 10 mm and used for lipid extraction. Unbound ceramides (A, N, O, and EO ceramides) and protein-bound ceramides (P–O ceramides) were extracted and their species with the C18 long-chain base were analyzed using an ultra-performance LC coupled with a triple quadrupole mass spectrometer Xevo TQ-S (Waters, Milford, MA) as described previously (5). The top 100 unbound ceramide species (14 classes) and the top 30 protein-bound ceramide species (five classes), which covered more than 95% of the total amount for each of these categories, were quantified by calculating the ratio of the peak area for each ceramide species to that of the internal standard corresponding to each ceramide class (18).

### Cell-based ULCFA ω-hydroxylase assay

ULCFA ω-hydroxylase assay was performed as previously described (9). The plasmids encoding human *ELOVL4* (pCE-puro 3×FLAG-ELOVL4), *CERS3* (pCE-puro 3×FLAG-CERS3), and *CYP4F22* (pCE-puro 3×FLAG-CYP4F22) were used for the expression of N-terminal 3×FLAG-tagged proteins. Plasmids encoding *CYP4F22 E79*∗ ((p.(Glu79∗)) or *R397C* (p.(Arg397Cys)) were generated using the pCE-puro 3×FLAG-CYP4F22 plasmid as a template, appropriate primers (*E79*∗, 5′-CATGTACCTTCCAAATTAGGCGGGCCTTCAAG-3′ and 5′-CTTGAAGGCCCGCCTAATTTGGAAGGTACATG-3′; *R397C*, 5′-CATTAAGGAGAGCCTGTGCCAGTACCCACCTG-3′ and 5′-CAGGTGGGTACTGGCACAGGCTCTCCTTAATG-3′), and the QuikChange Site-Directed Mutagenesis Kit (Agilent Technologies). HEK 293T cells were transfected with plasmids encoding *3×FLAG-ELOVL4*, *3×FLAG-CERS3*, and *3×FLAG-CYP4F22* (wild-type or mutant, E79∗ or R397C). Twenty-one hours after transfection, the cells were incubated for 30 min in a medium without fetal bovine serum and for another 3 h in a medium containing 1 μM seven deuterium (*d*_7_)-labeled sphingosine (Avanti Polar Lipids, Birmingham, AL) and 50 μM linoleic acid. The cells were washed twice with PBS and were collected in plastic tubes. After centrifugation (400 *g*, room temperature, 3 min), the cells were suspended in 100 μl of water and mixed with 375 μl of chloroform/methanol/12 M formic acid (100:200:1, vol/vol). As an internal standard, 2 pmol of *N*-(2′-(*R*)-hydroxypalmitoyl(*d*_9_))-D-*erythro*-sphingosine (*d*_9_-C16:0 AS, Avanti Polar Lipids) was added. Samples were then mixed with 125 μl of chloroform and 125 μl of water and centrifugated (20,400 *g*, room temperature, 3 min). The organic phase (lower phase) was collected, dried, and dissolved in 125 μl of chloroform/methanol (1:2, vol/vol). The products of CYP4F22, *d*_7_-ω-hydroxyceramides (*d*_7_-OS), were detected via LC/MS/MS (supplemental Table S1) and were quantified by calculating the ratio of the peak area of each *d*_7_-ω-hydroxyceramide species to that of the *d*_9_-C16:0 AS.

## Results

### Clinical features of the two SHCB cases

**Fig. 1:**
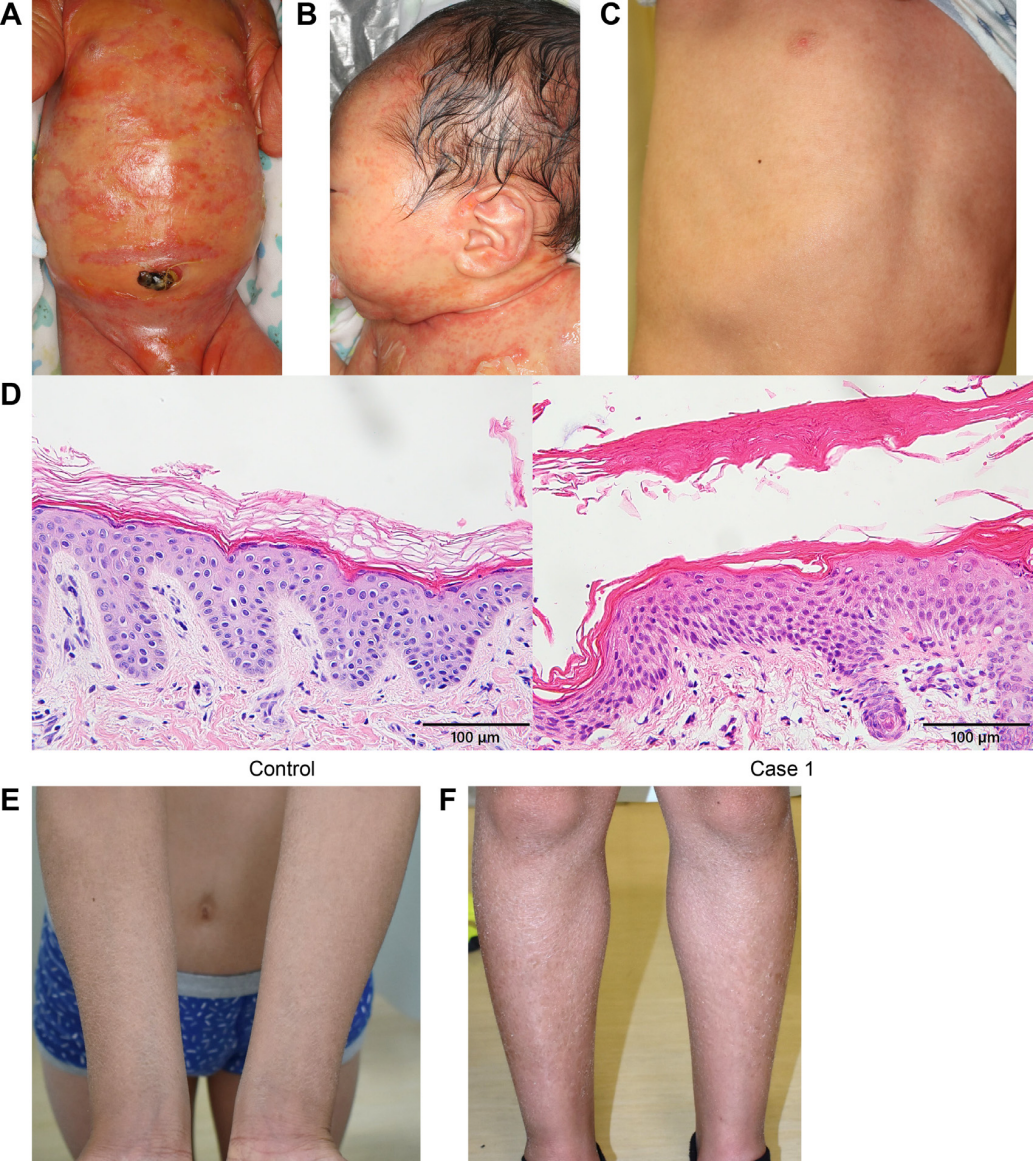
Clinical and histological features of two patients with SHCB. A–D, case 1. A: At 6 days of age, there are diffuse erythematous lesions and erosions with large scales on the chest and abdomen. B: At 10 days of age, erythematous hyperkeratosis on the face and neck and an erosion on the upper chest are evident. C: At 2 years of age, mild whitish scales on the chest are observed. D: A biopsy sample from the ichthyotic skin of case 1 shows compact hyperkeratosis, which suggests a deficiency of the intercellular lipid layers in the stratum corneum. The granular layers seen in the epidermis of case 1 are thinner than those of a healthy skin sample. Scale bars = 100 μm. E, F: case 2. Mild hyperkeratosis with fine, whitish scales is seen on the extensor surfaces of the arms (E) and legs (F). SHCB, self-healing collodion baby.

Case 1 was the first child born to nonrelated parents without any family history of similar disorders. He was born at full term after an uneventful pregnancy, with a birth weight of 2,938 g. The Apgar Score was 8/10 and 9/10 at 1 and 5 min, respectively. On examination, at birth, he showed a collodion membrane over his entire body surface, with moderate fissuring at the joints (Fig. 1A). During the first 10 days of life, the thick scales gradually desquamated (Fig. 1B). Cultures for microorganisms from skin samples detected methicillin-resistant *Staphylococcus aureus*. Intravenous vancomycin decreased the skin redness and erosions. His hair, nails, and teeth were normal. He had neither neurological symptoms nor hearing loss.

A skin biopsy specimen at 10 days after birth showed hyperkeratosis with thinned granular layers (Fig. 1D). Granular degeneration was not observed. Laboratory tests showed mild hyper-eosinophilia. At 2 years of age, he showed only extremely mild generalized ichthyosis and overlying mild fine scaling (Fig. 1C).

Case 2 is a 11-year-old male and the third child of nonrelated healthy parents. At the age of 12 months, he was diagnosed with congenital ichthyosis and he received emollients. His aunt and cousin also have ichthyoses. Clinical examinations found very mild fine scales on the trunk and extremities (Fig. 1E, F). Case 1 and case 2 were from independent families.

### Mutation detection

To identify the underlying molecular genetic defects in both patients, we obtained blood samples from the two patients and their parents for genetic testing. Based on the mild phenotypes and high prevalence of common ichthyoses (11), we suspected that the diagnosis for the two patients was ichthyosis vulgaris caused by *FLG* mutations or recessive X-linked ichthyosis associated with *STS* (steroid sulfatase) deletions. Initial fluorescence in situ hybridization analysis for Xp22.3, which includes the region of *STS* on chromosome X, revealed no deletion of Xp22.3 in either patient. Next, the genotyping of the *FLG* mutations, all of which are loss-of-function mutations, was performed with the TaqMan probe genotyping assay. In neither patient did we identify any putative pathogenic mutation in *FLG*.

**Fig. 2:**
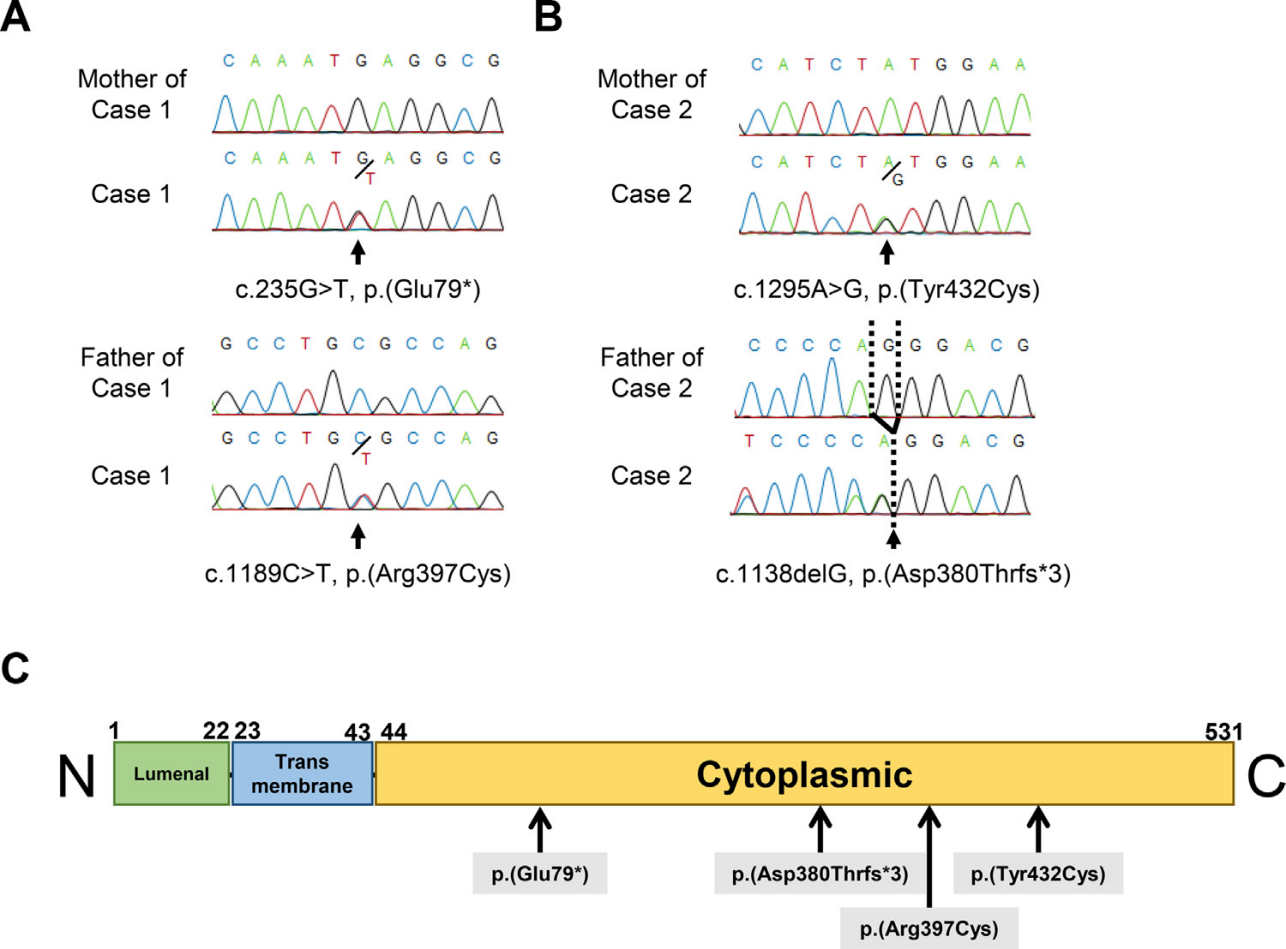
*CYP4F22* mutations detected in the present two patients with SHCB and the domain structure of CYP4F22. A, B: Sanger sequencing confirms *CYP4F22* mutations in genomic DNA. Chromatograms illustrate the four *CYP4F22* mutations identified in this study. A: case 1 is compound heterozygous for the c.235G>T, p.(Glu79∗) and c.1189C>T, p.(Arg397Cys) mutations. B: case 2 is compound heterozygous for c.1295A>G, p.(Tyr432Cys) and c.1138delG, p.(Asp380Thrfs∗3). C: The CYP4F22 domain structure, with the mutations indicated. The mutations in case 1 and case 2 are marked by black arrows. SHCB, self-healing collodion baby.

Then, WES was performed for case 1, his parents, and case 2. The filtered rare variant list generated from the whole-exome data showed the two affected individuals to harbor rare or novel compound heterozygous mutations in *CYP4F22*. These mutations were verified by Sanger sequencing (Fig. 2A, B) and were confirmed to segregate with disease status in family members whose DNA was available. WES failed to reveal any pathogenic mutations in other genes known to be implicated in ichthyosis. Pathogenic variants of *CYP4F22* are reported to be scattered throughout the *CYP4F22* gene, and most of the pathogenic *CYP4F22* mutations are located within the longest cytoplasmic domain of the CYP4F22 protein (www.hgmd.cf.ac.uk, Human Gene Mutation Database Professional, as of 2021.4) (13). The present four mutations lie in the CYP4F22 cytoplasmic domain (Fig. 2C). The mutation c.235G>T, p.(Glu79∗) has not been described in the gnomAD database (19) nor in the dbSNP database. The global allele frequencies of c.1189C>T, p.(Arg397Cys) (rs572771583) and c.1295A>G, p.(Tyr432Cys) (rs1430532183) are 0.000003976 (one heterozygous carrier was reported for each mutation in the gnomAD database (19)). Several protein function prediction browsers (e. g., SIFT (20), PolyPhen-2 (21), MutationTaster (22)) attributed very high scores for likelihood of damage.

### Immunohistochemical analysis of the skin specimen from case 1

**Fig. 3:**
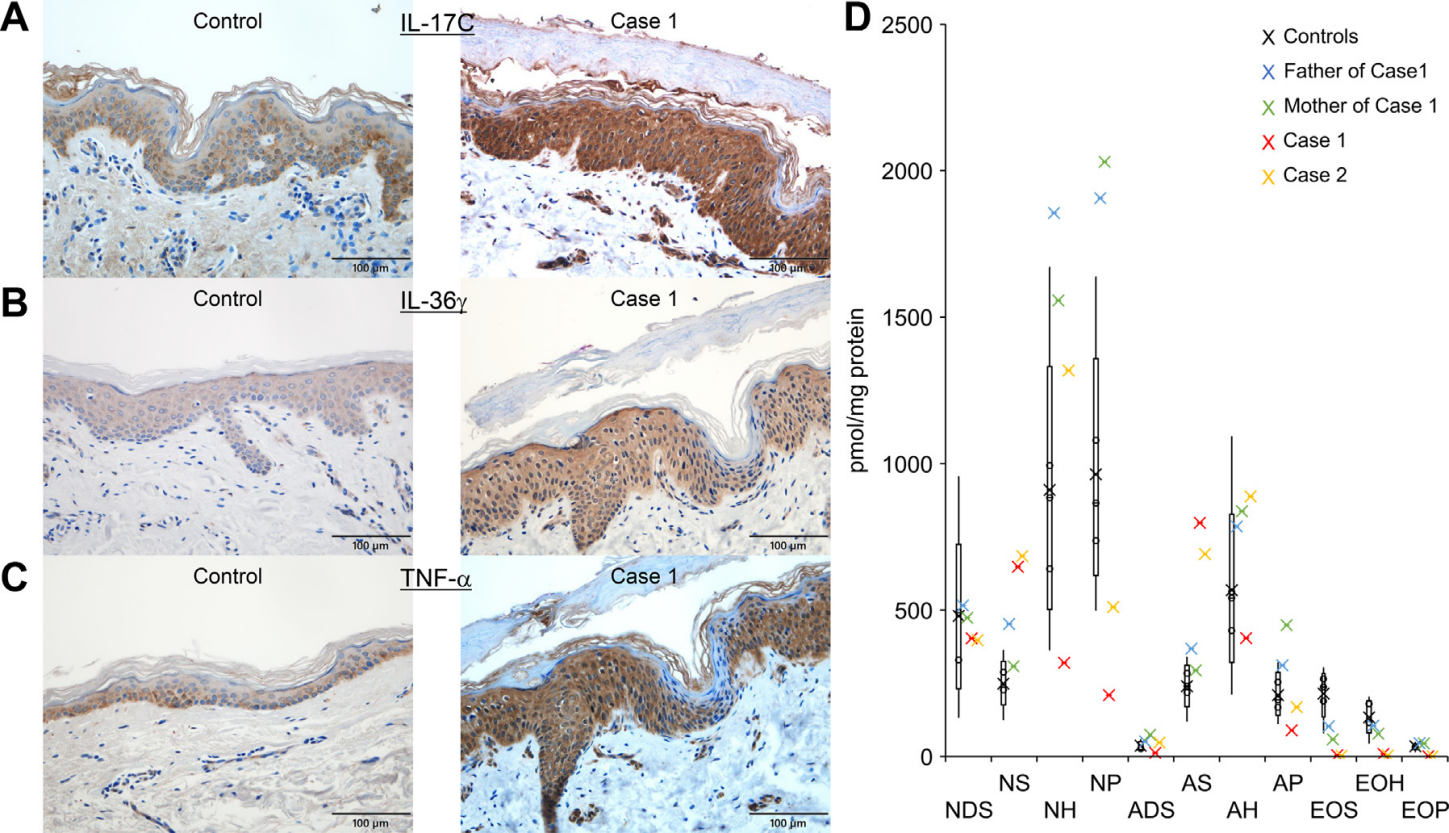
Expression of IL-17C, IL-36γ, and TNF-α in SHCB skin lesions and analysis of ceramide components in the patients’ stratum corneum. A–C: Skin samples from case 1 (right) and from healthy control donors (left) were stained with anti-IL-17C (A), anti-IL-36γ (B), and anti-TNF-α (C) antibodies. Scale bars: 100 μm. D: Amounts of ceramide from each class in the stratum corneum of both patients, the parents of case 1 and controls. SHCB, self-healing collodion baby.

We conducted immunohistochemical analysis with anti-IL-17C, anti-IL-36γ, and anti-TNFα antibodies of a lesional skin sample from case 1 to assess whether cutaneous inflammation occurred. The staining intensities of IL-17C (Fig. 3A), IL-36γ (Fig. 3B), and TNF-α (Fig. 3C) were significantly greater in the patient’s skin than in the skin from a healthy control.

### Ceramide profiles in the stratum corneum of the patients

The levels of unbound and protein-bound ceramides in the tape-stripped skin samples from the right leg were examined by LC/MS/MS. Although the overall effects of the bi-allelic mutations in *CYP4F22* on the levels of total ceramides were small, it is notable that the levels of acylceramides (EOS, EOH, and EOP) and protein-bound ceramides (P-OS and P-OH) were much lower in the stratum corneum of the patients than in the unaffected parents and normal controls (Fig. 3D and Table 1, Table 2, Table 3). Additionally, the amounts of NP and AP were lower in the stratum corneum of the patients than in the controls. In contrast, the levels of NS and AS were elevated in the patients’ samples. Regarding FA composition, shortening of nonacylated ceramides (e. g., NH) was observed (Table 4). In patients, NH species with ≥C26 FAs were reduced compared to healthy subjects, but instead C16–C22 species were increased.

**Table 1:** Amount of each ceramid class in the stratum corneum (pmol/mg protein)

Ceramide Classes	Average of Controls (n = 5)	Father of Case 1	Mother of Case 1	Case 1	Case 2
NDS	481	516	474	404	398
NS	249	453	309	648	684
NH	910	1855	1557	320	1317
NP	964	1906	2030	209	510
ADS	36	51	75	12	48
AS	241	368	294	798	691
AH	568	786	837	405	888
AP	210	311	449	89	168
EOS	215	103	59	4	2
EOH	133	105	77	9	4
EOP	35	45	45	1	1
OS	27	19	13	4	4
OH	13	7	7	2	0
OP	8	6	5	5	2
Total	4089	6530	6230	2910	4718

**Table 2:** Percentages of ceramide classes in the stratum corneum

Ceramide Classes	Averages of Controls (n = 5)	Father of Case 1	Mother of Case 1	Case 1	Case 2
NDS	12	7.9	7.6	13.9	8.4
NS	6.3	6.9	5	22.3	14.5
NH	21.6	28.4	25	11	27.9
NP	23.7	29.2	32.6	7.2	10.8
ADS	0.9	0.8	1.2	0.4	1
AS	6.1	5.6	4.7	27.4	14.7
AH	13.3	12	13.4	13.9	18.8
AP	5.3	4.8	7.2	3.1	3.6
EOS	5.4	1.6	0.9	0.1	0.1
EOH	3.3	1.6	1.2	0.3	0.1
EOP	0.9	0.7	0.7	0	0
OS	0.7	0.3	0.2	0.2	0.1
OH	0.3	0.1	0.1	0.1	0
OP	0.2	0.1	0.1	0.2	0.1

**Table 3:** Amount of protein-bound ceramide in the stratum corneum (pmol/mg protein)

Ceramide Classes	Average of Controls (n = 5)	Father of Case 1	Mother of Case 1	Case 1	Case 2
P-OS	113	93	165	0	37
P-ODS	0	0.1	0.8	0	0
P-OH	31	47	88	0	5
P-OP	2	3.8	6.7	0	0.1
P-OSD	2	2	2.5	0	0.5

**Table 4:** Quantities of total NH and each NH species and percentages of each NH species

NH Species of Different Carbon Length	Average of Controls (n = 5)		Father of Case 1		Mother of Case 1		Case 1		Case 2	
	pmol/mg Protein	Percentage (%)	pmol/mg Protein	Percentage (%)	pmol/mg Protein	Percentage (%)	pmol/mg Protein	Percentage (%)	pmol/mg Protein	Percentage (%)
C16:0	11	1.4	13.2	1.2	21.7	0.8	17	5.3	71.4	5.4
C18:0	9.2	1	4.5	0.4	7.7	0.3	22.5	7	43	3.3
C20:0	6.6	0.8	5.3	0.3	5	0.3	13.6	4.2	22	1.7
C22:0	19	2.1	17.8	1	19.4	1.1	23.1	7.2	63	4.8
C23:0	20.7	2.3	20.1	1.3	24.3	1.3	14.5	4.5	38.2	2.9
C24:0	213.1	22.8	308.8	19.3	358.9	19.8	101	31.5	299.5	22.7
C25:0	86.8	9.2	170.6	10.3	190.6	11	28	8.7	95.7	7.3
C26:0	318	34.8	652.2	39.7	737.2	41.9	73.5	23	362.9	27.5
C27:0	52.4	5.6	86.6	6.3	116.8	5.6	3.3	1	59.3	4.5
C28:0	137.5	15.7	217.4	15.8	292.5	14	13.8	4.3	181.7	13.8
C29:0	16.6	1.9	24.7	1.7	32.1	1.6	4.5	1.4	25.7	2
C30:0	19.3	2.3	35.8	2.6	49	2.3	5.5	1.7	54.7	4.2
Total	910.2	100	1556.9	100	1855.1	100	320.2	100	1317.3	100

### *CYP4F22* mutations impair enzymatic activity

**Fig. 4:**
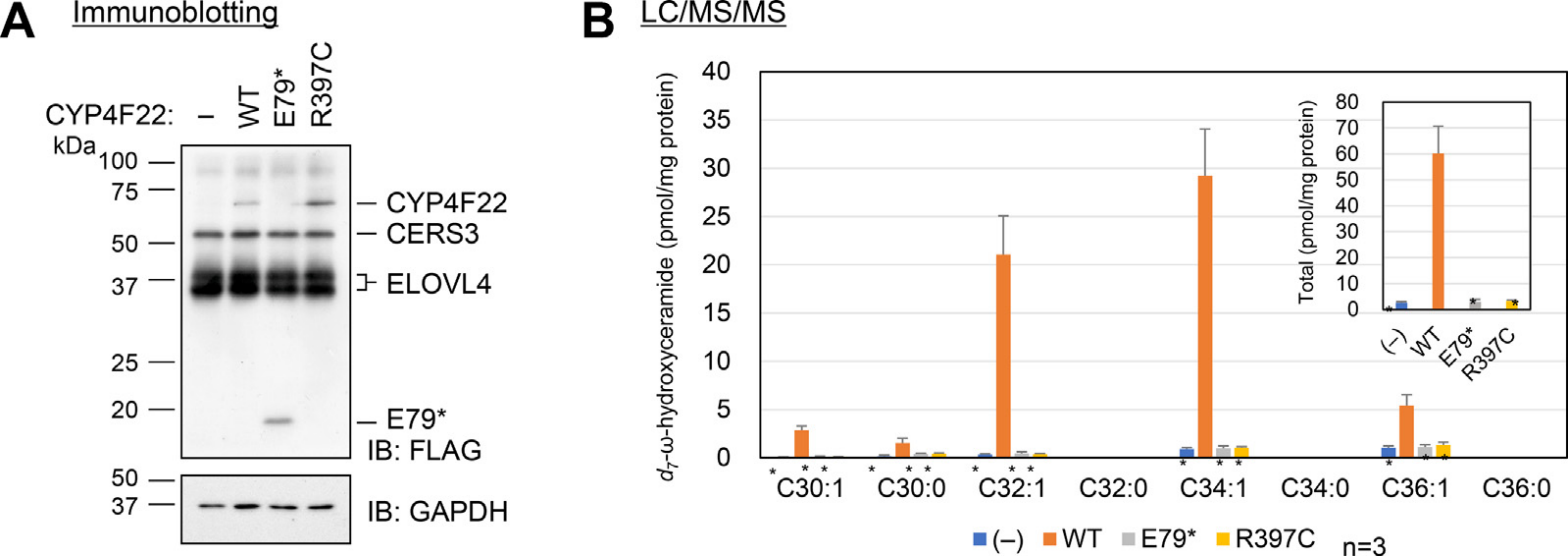
Mutations, p.(Glu79∗) and p.(Arg397Cys), in *CYP4F22* impair enzymatic activity. HEK 293T cells were transfected with the pCE-puro 3×FLAG-1 (vector) or pCE-puro 3×FLAG-CYP4F22 (wild type [WT], E79∗, or R397C mutant) plasmid, together with pCE-puro 3×FLAG-ELOVL4 and pCE-puro 3×FLAG-CERS3 plasmids and were incubated with 1 μM *d*_7_-sphingosine and 50 μM linoleic acid for 3 h. A: Total cell lysates (5 μg) were separated by SDS-PAGE and subjected to immunoblotting using anti-FLAG antibody (upper panel) or anti-GAPDH antibody (lower panel, loading control). E79∗, p.(Glu79∗); R397C, p.(Arg397Cys). B: Amounts of *d*_7_-ω-hydroxyceramide species with saturated and monounsaturated FAs (C30–36) were quantified by LC/MS/MS analyses. Values represent the mean + standard deviations of three independent experiments (n = 3, ∗∗ *P* < 0.01; Dunnett’s test; vs. WT). IB, immunoblotting.

To assess the effect of the mutations on CYP4F22 enzymatic activity, a cell-based assay was conducted, where wild type or mutants (p.Glu79∗ and p.Arg397Cys) of CYP4F22 were overproduced, together with the FA elongase ELOVL4 and the ceramide synthase CERS3 (all tagged with 3×FLAG). Expression of these proteins was confirmed by immunoblotting (Fig. 4A). Quantification of the CYP4F22 products ω-hydroxy ceramides (OS) by LC/MS/MS revealed that the OS levels in cells expressing either mutant was comparable to those in the vector-transfected cells (Fig. 4B), indicating that both mutants have deficient enzyme activity.

## Discussion

We previously reported that IL-17C and IL-36 family cytokines were upregulated in the skin of an adult ARCI patient with a *NIPAL4* mutation (23). In the literature, the significant upregulation of IL-17/TNF-α-related genes and psoriasis hallmark genes has been reported in various ARCI patients (24). Malik *et al.* reported that IL-17-associated markers in ichthyotic skin and serum IL-17A levels cluster tightly with disease severity (ichthyosis area and severity index–erythema), whereas epidermal dysfunction (transepidermal water loss) correlate most closely with *EREG* and *IL36B* mRNA expression levels (24). However, patients with SHCB caused by *CYP4F22* mutations were not included in their study. Our immunohistochemical staining results indicate that there was certain cutaneous inflammation with the strong expression of IL-17C, IL-36γ, and TNF-α in case 1 at birth (Fig. 3). Although skin samples from SHCB patients after self-healing were unavailable, the initial inflammation in the SHCB patients due to barrier dysfunction is expected to abate with age. Thus, during the neonatal period, the immune profiles might be shared between SHCB and other ARCIs, and it is not easy to distinguish SHCB from other forms of ARCI, such as CIE.

LC/MS/MS measurement in this study revealed decreases in the amounts of acylceramides (EO ceramides), protein-bound ceramides (P-O ceramides), and P-type ceramides (NP and AP) in the SHCB patients (Fig. 3D and Table 1, Table 2, Table 3). Acylceramides are thought to stabilize lipid lamellae by interconnecting the layers of the lamellae (25). Protein-bound ceramides (CLE) may function to connect lipid lamellae and corneocytes. The 4-hydroxyl group in the long-chain base moiety of P-type ceramides enhances the lipid-lipid interaction in lipid lamellae through hydrogen bonding. NP levels are decreased in patients with atopic dermatitis and are inversely correlated with the values of transepidermal water loss in healthy subjects in addition to the patients (26, 27). Thus, all of the ceramide classes reduced in the SHCB patients in this study are important for lipid lamella or CLE formation. The increased amounts of AS and NS would be primarily due to compensation for the decrease in P-type ceramides.

Interestingly, although very low levels of acylceramides and protein-bound ceramides were found in the stratum corneum, both patients showed mild to moderate phenotypes of congenital ichthyosis. We speculate that these rather mild phenotypes from *CYP4F22* mutations might be caused by mild inflammation in the skin. Clinically, the cutaneous inflammation in patients with *KDSR* (3-Ketodihydrosphingosine Reductase) mutations, *PHGDH* (Phosphoglycerate Dehydrogenase) mutations, and *NIPAL4* mutations is more severe than in patients with *SDR9C7* mutations (2, 23, 28, 29). This difference is also histologically confirmed in skin biopsy samples from the patients. Additionally, microarray gene expression profiling by using *Sdr9c7* knockout mice indicated that the gene expression changes of skin-associated immune responses in the skin from *Sdr9c7* knockout mice were very limited compare with those changes in wild-type mice (1). The severity of inflammation modifies the phenotype in each patient with ichthyosis and, clinically, anti-inflammatory therapies (e. g., anti-TNF-α, anti-IL-17, and anti-IL-4/IL-13 antibodies) have been reported as useful treatments for several types of inherited ichthyoses (30, 31).

The present study revealed a novel mutation in *CYP4F22*, c.1295A>G, p.(Tyr432Cys), in case 2, and three previously reported mutations: c.235G>T/p.(Glu79∗), c.1189C>T/p.(Arg397Cys), and c.1138delG/p.(Asp380Thrfs∗3) (9, 15, 32) (Fig. 2). The nonsense mutation p.(Glu79∗) causes the complete loss of enzyme activity for CYP4F22 (Fig. 4B). The frameshift mutation c.1138delG in *CYP4F22* is predicted to cause truncation of the CYP4F22 p.(Asp380Thrfs∗3) protein and to lead loss of enzyme activity. The missense mutation p.(Arg397Cys) changes a basic amino acid into a neutral, polar one in the cytoplasmic domain of the molecule. As for p.(Tyr432Cys), both tyrosine and cysteine are neutral, polar amino acids, but unlike cysteine, tyrosine is an aromatic acid. Several protein function prediction browsers including SIFT, PolyPhen-2, and MutationTaster attribute very high scores for likelihood of damage to both substitutions. Indeed, we confirm that p.(Arg397Cys) loses activity (Fig. 4B).

The present probands showed SHCB, a relatively mild phenotype of ARCI. At present, genotype/phenotype correlations in ARCI that are associated with *CYP4F22* mutation are uncertain. Both of the present patients are compound heterozygous for missense and truncating mutations in *CYP4F22*. However, there are a few differences in the stratum corneum ceramide profiles between the present two SHCB patients. In the literature, there are several reports of homozygous or compound heterozygous missense mutations in *CYP4F22* leading not only to SHCB but also to lamellar ichthyosis, a nonimproving ARCI phenotype (33, 34). There are no obvious differences in the results of the present cell-based ω-hydroxylase assay between missense and truncating mutations in *CYP4F22*. Thus, whether causative mutations are missense or truncating might not be a definitive factor for the SHCB phenotype in patients with ARCI from *CYP4F22* mutations. Concerning the mechanism of “self-healing” in the present patients, several possibilities are conceivable, including the normalization of ceramide profiles with improvements in the patient’s phenotype and compensation for the role of CYP4F22, ω-hydroxylation of the ceramides by another cytochrome p450 enzyme. However, in our present results, the ceramide profile did not normalize with improvements in the patients. Furthermore, we previously reported that the production of ω-hydroxy ceramides is below detectable levels in HEK293T cells that overexpress ELOVL4, CERS3, and CYP4F11 (9). Thus, it is unlikely that CYP4F11 would take over the role of ω-hydroxylating the ceramides. We would like to accumulate and analyze additional SHCB patients with *CYP4F22* mutations in order to clarify the mechanism of self-healing.

There are unique and characteristic features of the ceramide composition in the stratum corneum of patients with syndromic or nonsyndromic ichthyosis, depending on the causative genes. Genetic diagnosis using next-generation sequencing, such as WES, sometimes detects many variants of unknown significance and it is very difficult to determine the truly pathogenic variants. Thus, well-established in vitro or in vivo functional studies are frequently needed to determine the pathogenicity of novel mutations (35). The work involved, such as a cell-based assay using mutant plasmids (32), is often time-consuming, labor-intensive, and expensive. In this context, detailed analyses of ceramides in the stratum corneum of patients with ichthyosis might provide valuable clues for detecting causative genes in each patient. WES coupled with noninvasive ceramide analyses using stratum corneum samples obtained by tape stripping is useful for clinical diagnosis, especially for patients with ichthyosis in early infancy. In the Middle-Eastern population, higher prevalences of *CYP4F22* and *ABCA12* pathogenic variants and lower prevalences of *TGM1* and *NIPAL4* variants have been reported, as compared with data obtained in other regions of the world (15). Perhaps the present results from noninvasive ceramide analyses are more valuable for patients with ARCI in the Middle-Eastern population.

In conclusion, our findings, in combination with previous reports, suggest that WES coupled with noninvasive ceramide analyses using stratum corneum samples obtained by tape stripping might be useful for the early and precise diagnosis of congenital ichthyosis.

## Data Availability

All data are contained within the article.

## Supplemental Data

This article contains supplemental data.

## Conflicts of Interest

The authors declare that we have no conflicts of interest.
