# Supplementary material for: Ceramide Analysis in Combination With Genetic Testing May Provide a Precise Diagnosis for Self-Healing Collodion Babies
Source: J Lipid Res. 2022 Nov 1;63(12):100308. doi: 10.1016/j.jlr.2022.100308 (PMC9703634; doi:10.1016/j.jlr.2022.100308)
Supplement: Supplemental data [file mmc1.pdf]

Ceramide analysis in combination with genetic testing may provide a precise diagnosis for self-healing collodion babies

Takeichi et al.

Supplemental file

Supplemental Figure pp. 2

Figure S1. Nomenclature of ceramide classes.

Supplemental Table pp. 3

Table S1. MRM settings for detection of d7-ω-hydroxyceramide (d7OS) species

Figure S1. Nomenclature of ceramide classes.

FA / Long-chain Base	**N**	**A**	**O**	**EO**	**P-O**
DS	**N**DS	**A**DS	**O**DS	**EO**DS	**P-O**DS
S	**N**S	**A**S	**O**S	**EO**S	**P-O**S
P	**N**P	**A**P	**O**P	**EO**P	**P-O**P
H	**N**H	**A**H	**O**H	**EO**H	**P-O**H
SD	**N**SD	**A**SD	**O**SD	**EO**SD	**P-O**SD

Nonacylated ceramides   Acylceramides

Free (non-protein-bound) ceramides   Protein-bound ceramides

Each ceramide class is represented by a combination of the abbreviations for its constituent long-chain base and FA. Ceramides are classified into free ceramides (non-protein-bound ceramides) and protein-bound ceramides (P–O ceramides), and the former are subclassified into non-acylated ceramides (N, A, and O ceramides) and acylceramides (EO ceramides). N: non-hydroxy FA, A: α-hydroxy FA, O: ω-hydroxy FA, EO: esterified ω-hydroxy FA, P–O: protein-bound ω-hydroxy FA. DS: dihydrosphingosine, S: sphingosine, P: phytosphingosine, H: 6-hydroxysphingosine, SD: 4,14-sphingadiene.

Table S1. MRM settings for detection of d7-ω-hydroxyceramide (d7OS) species.

Ceramide Species	Precursor ions (Q1)		Product ion (Q3)	Collision energy (eV)
	$[M–H_2O+H]^+$	$[M+H]^+$		
d_7OS (C30:1)	737.7	755.7	271.3	35
d_7OS (C30:0)	739.7	757.7	271.3	35
d_7OS (C32:1)	765.7	783.7	271.3	35
d_7OS (C32:0)	767.7	785.7	271.3	35
d_7OS (C34:1)	793.7	811.7	271.3	40
d_7OS (C34:0)	795.7	813.7	271.3	40
d_7OS (C36:1)	821.7	839.7	271.3	40
d_7OS (C36:0)	823.7	841.7	271.3	40
